# Low-Protein Diet Supplemented with Amino Acids Can Regulate the Growth Performance, Meat Quality, and Flavor of the Bamei Pigs

**DOI:** 10.3390/foods14060946

**Published:** 2025-03-11

**Authors:** Dong Wang, Ke Hou, Mengjie Kong, Wei Zhang, Wenzhong Li, Yiwen Geng, Chao Ma, Guoshun Chen

**Affiliations:** College of Animal Science and Technology, Gansu Agricultural University, Lanzhou 730070, China; wangd@st.gsau.edu.cn (D.W.);

**Keywords:** Bamei pig, dietary protein levels, amino acid supplementation, growth performance, meat quality, meat flavor

## Abstract

This study evaluated the impact of reduced crude protein (CP) diets supplemented with four essential amino acids (EAAs) on production efficiency and meat quality characteristics in Bamei pigs. Thirty-six castrated Bamei pigs (half male and half female, 100 days old, with an average body weight of 50.65 kg) were randomly assigned to three different dietary CP levels: control group (16.0% CP), group I (14.0% CP + EAA), and group II (12.0% CP + EAA). In both experiments, the group I and group II diets were supplemented with crystalline AA to achieve equal contents of standardized ileal digestible (SID) lysine, methionine, threonine, and tryptophan. After a 70-day feeding trial, the results showed that (1) low-protein diets of different levels supplemented with four EAAs had no significant effect on the growth performance of Bamei pigs (*p* > 0.05) but had a tendency to increase average daily feed intake (ADFI). (2) In terms of slaughter performance, compared with the control group, the low-protein amino-acid-balanced diet significantly reduced the pH of gastric contents (*p* = 0.045), and tended to increase the backfat thickness and dressing percentage (*p* > 0.05). (3) The effect of low-protein diets on muscle amino acids showed that group I was significantly improved, including increased Threonine, Serine, Glycine and Bitter amino acids. (4) Compared with the control group, the low-protein group increased the ratio of unsaturated fatty acid (UFA)/total fatty acids (TFAs), Monounsaturated Fatty Acid (MUFA)/TFA, and Polyunsaturated Fatty Acid (PUFA)/TFA, and the content of decanoic acid, myristic acid, and cis-11-eicosenoic acid in group II was significantly higher than that in the other two groups (*p* ≤ 0.012). (5) The total number of flavor compounds in the muscle of the low-protein group was higher than that of the control group, including Aldehyde, Alcohol, sulfide, Alkane, and Furan compounds. Among them, the relative contents of Hexanal, Heptaldehyde, Benzaldehyde, E-2-Octenal, 2,3-Octanedione, and 2-Pentylfuran in group II were significantly higher than in those groups (*p* < 0.05). Notably, the 14% dietary protein level group had the most significant effect on the meat quality and flavor of Bamei pigs. Therefore, under the condition of amino acid balance, reducing the use of protein feed raw materials and adding synthetic amino acids can not only improve the meat quality and flavor of finishing pigs, but also save the feed cost.

## 1. Introduction

Low-protein diets are a nutritional strategy designed to reduce the protein content in pig feed from 2.0% to 4.0%, while supplementing EAA tailored to the specific growth stages of pigs, based on the protein recommendations outlined by the NRC 2012 [1]. Low-protein diets have garnered significant attention within the pig industry due to their numerous advantages, including the conservation of protein resources, enhanced feed utilization, reduced feed costs, and mitigation of environmental pollution [2,3,4,5]. Previous studies have shown that low-protein diets have no significant effect on the growth performance of fattening pigs [6]. Some studies have found that the backfat thickness and eye muscle area of fattening pigs are not affected by the change in feed protein level (14.0–17.9% CP) [7,8]. However, feeding a low-protein diet has been found to significantly increase intramuscular fat (IMF), Monounsaturated Fatty Acid (MUFA) content, and the a* value in fattening pigs [9]. Furthermore, some studies have reported that low-protein diets enhance the a* value and reduce muscle shear force [10]. In recent years, the Chinese pork market has predominantly featured ternary hybrid pigs (Duroc × Landrace × Yorkshire), with a limited presence of high-quality pork derived from local varieties [11]. The Bamei pig, an esteemed local breed in China, holds significant potential for producing high-quality pork and plays a crucial role in the development of new pig varieties [12,13]. Current research by our group has revealed that, under the condition of SID AA balance, reducing the dietary protein level of fattening pigs by 2.0% to 4.0% does not significantly impact their growth and slaughter performance. However, it enhances the number and content of volatile flavor substances in muscle, thereby positively influencing meat quality [14]. Considering the multifaceted benefits associated with amino-acid-balanced low-protein diets, this study aimed to investigate the regulatory influence of varying protein levels on production performance, meat quality, and flavor profiles in Bamei pigs during the fattening period. The findings of this research are expected to contribute valuable data support for the comprehensive utilization and sustainable development of this exceptional local pig breed resource. Additionally, the results will provide a scientific foundation for the optimization of breeding strategies for Bamei pigs, thereby enhancing their agricultural and economic value.

## 2. Materials and Methods

### 2.1. Ethics Statement

The present study was approved by the Ethics Committee of the Gansu Agricultural University (GSAU-Eth-AST-2021-016).

### 2.2. Preparation of Diets

The preparation of the diets conforms to the low-protein feed standard for growing and fattening pigs proposed by the Standards and Technical Committee of the China Feed Industry Association Group in 2018, and refers to the results of previous studies on SID AA-balanced low-protein diets [15]. The composition and nutrient content of the feed are shown in Table 1.

### 2.3. Animals, Feeding Management, and Experimental Design

Thirty-six 100-day-old (average body weight 50.65 kg) Bamei pigs (boars were castrated pigs) were randomly allotted to 3 different dietary treatment groups according to gender (half male and half female), with 12 pigs in each group. Each group was divided into 3 replicates with 4 pigs in each replicate. In the three dietary treatments, the control group (16.0% CP), group I (14.0% CP + EAA), and group II (12.0% CP + EAA) were used. Four crystalline amino acids (lysine, methionine, threonine, and tryptophan) were added to the diets of group I and group II to ensure that the content of SID AA in the three experimental diets was equal. The experiment lasted for 80 days, including a 10-day adaptation period and a 70-day experiment period. During the feeding period, pigs were fed according to the repetition of each group in a column feeding system. These pigs were raised on a farm named Gansu Agricultural and Animal Husbandry Breeding Farm in Gansu Province of China. The pig house was cleaned and disinfected before the study. During the study, pigs were dewormed and immunized, according to standard pig farm management protocols. Diets were provided at 8:00 and 18:00 every day, and the pigs had free access to feed and water. The pig house cleaned twice daily and disinfected once a week. During the test period, the amount of feed given and the amount remaining in each group were recorded every day, and the daily feed intake was calculated.

### 2.4. Growth Performance and Measurement Methods

All experimental pigs were weighed in units of repetition after fasting for 24 h on the 0th and 70th days of the formal experimental period. Free drinking water was allowed, and the initial body weight (IBW) and final body weight (FBW) of each column were recorded, and the average daily gain (ADG) was calculated. The diet consumption of each group of pigs during the experiment was accurately recorded, and the average daily feed intake (ADFI) and feed conversion ratio (FCR) were calculated.

ADG = (FBW − IBW)/experimental days

ADFI = total feed intake/(feeding days × number of feeding)
FCR = total feed intake (kg)/total weight gain (kg)

### 2.5. Slaughtering Performance and Measurement Methods

After a 70 day experimental period, feeding was stopped, the pigs were fasted for 24 h, free drinking water was allowed, and 2 pigs (one male and one female) were randomly selected from each replicate in each group for slaughtering. Specifically, the experimental pigs were exsanguinated after being stunned according to the “Good Practice for the Slaughtering of Livestock and Poultry-Pigs” (Chinese Standard GB/T 19479-2019). The carcass weight, carcass length, and average backfat thickness were measured according to the method provided by Wang et al. [14]. The loin eye area was measured according to the method of Li et al. [16]. The heart, liver, spleen, lung, kidney, and hind leg were separated and weighed, and then the pH of gastric contents and intestinal contents (jejunum and cecum) were measured, and then the gastric contents were emptied and weighed. The pH of gastric and intestinal contents was measured using a portable pH meter (CP-461, Elmetron, Zabrze, Poland). Following the American Meat Science Association (AMSA) sampling guidelines, the longissimus thoracis muscle spanning the 4th to 6th thoracic vertebrae of the left carcass from experimental pigs was aseptically excised post-mortem after slaughter. The excised muscle was vacuum-sealed in oxygen-barrier packaging and stored under conditionally optimized preservation protocols pending subsequent analyses.

### 2.6. Determination and Method of Pork Physical Quality

The determination method of shear force (hardness) referred to NY/T 1180-2006 “Determination of Meat Tenderness—Determination of Shear Force”. The instrument for measuring the shear force was the GR-150 texture analyzer (G&R Manufacturing, Thousand Oaks, CA, USA) equipped with a V-notch blade (1.27 mm thickness), and the following detection steps were performed: The whole meat sample with a length × width × height of not less than 6 cm × 3 cm × 3 cm was taken, and the tendons, fascia, and fats on the surface of the meat were removed. The meat sample was placed in a constant temperature water bath with a power of 1500 W, heated to a central temperature of 70 °C, and then removed and cooled to a central temperature of 0–4 °C. Cylindrical meat cores measuring 1.27 cm in diameter and ≥2.5 cm in length were excised parallel to the muscle fiber orientation using a circular sampler. To ensure structural integrity, cores were extracted at a minimum distance of 5 mm from the sample edges, with a spacing of ≥5 mm between adjacent cores to prevent mechanical interference. Cores exhibiting visible defects, such as voids or excessive fat marbling (>5% area), were discarded during quality control screening. Shear force measurements were performed using a texture analyzer configured with a blade speed of 225 mm/min and a trigger force threshold of 50 g. During testing, each core was mounted perpendicular to the blade trajectory, and the peak shear force (in Newtons, N) required to transect the core was recorded. Triplicate technical measurements were performed for each sample, with a minimum of three biological replicates (n ≥ 3) analyzed to ensure statistical robustness. The filtration rate (water retention capacity) was quantified using an RH-1000 Muscle filtration rate tester (Guangzhou Runhu Instruments Co., Ltd., Guangzhou, China) following the standardized protocol outlined in NY/T 1333-2007 Determination of Meat Quality of Livestock and Poultry. The procedure comprised the following steps: Fresh longissimus thoracis muscle sections (5.0 cm thickness) were transversely sliced into 1.0 cm thick specimens perpendicular to the muscle fiber orientation using a double-blade knife. Cylindrical cores (2.523 cm diameter) were excised from the anatomical mid-region of each slice using a circular sampler. To minimize moisture loss, the excised cores were immediately weighed (m_1_) with a high-precision analytical balance (±0.001 g resolution) within 30 s of sampling. The weighed meat sample was placed between 2 layers of gauze, and 8 layers and 6 layers of qualitative medium-speed filter paper were placed on the upper and lower layers of gauze. Then, the paper with the meat sample was moved to the table of the test base, and the midpoint of the base was aligned. The texture analyzer was pressurized to 350 N and kept for 300 s. After the pressure was removed, the meat sample was immediately stripped from the gauze and weighed as m_2_, and the filtration rate(%) was calculated as [(m_1_ − m_2_)/m_1_] × 100%. At the same time, it was ensured that the number of test samples was not less than 3. The cooking loss, marble score, and pH were measured according to the method of Skrlep et al. [17]. The values of meat color lightness (L*), redness (a*), and yellowness (b*) were measured by a CR-10 colorimeter (Konica Minolta, Osaka, Japan), with the following operational parameters: illuminant: D65 standard daylight source; observer angle: 10°; measurement aperture: 8 mm diameter; calibration: certified white ceramic tile (Y = 93.6, x = 0.3134, y = 0.3194); measurement mode: 3 consecutive readings per sample, averaged to minimize spatial heterogeneity; data output: L* (lightness), a* (redness) and b* (yellowness).

### 2.7. Determination and Method of Nutritional Value of Pork

The fresh longissimus thoracis muscle of pigs after slaughter was taken to determine the nutritional components of pork, following China’s national standards for determination. Determination of moisture and ash was performed following GB 5009.3-2016 (105 °C oven drying method). Determination of crude protein (CP) was performed following GB 5009.5-2016 (FOSS Kjeldahl semi micro Kjeldahl method). Determination of crude fat (EE) was performed following GB 5009.6-2016 (Soxhlet extraction method).

### 2.8. Determination of Content of Inosinic Acid and Related Substances in Pork

Inosinic acid (IMP) was measured in fresh longissimus thoracis muscle after slaughter. The determination of IMP was based on the T/NAIA 003-2020 (determination of inosine and inosini cacid in muscle high-performance liquid chromatography) and the method provided by Liu et al. [18]. Measurement was performed by means of LC-20A HPLC with an SPD-M20A UV Detector (Shimadzu Scientific Instruments, Inc., Kyoto, Japan). The detection steps were performed as follows: The muscle tissue was ground or crushed with liquid nitrogen, and 2 g (accurate to 0.0001 g) was weighed and placed in a 50 mL centrifuge tube. The homogenate was homogenized three times with 20 mL pre-cooled 5% perchloric acid solution. The homogenate was centrifuged at 4000 r/min for 10 min at 4 °C. The supernatant was transferred to a 50 mL beaker, and the supernatant was combined. The pH was adjusted to 6.5 with sodium hydroxide solution. The volume was adjusted to 50 mL with distilled water, shaken, filtered by a 0.45 μm filter membrane and loaded into the injection vial for testing. The chromatographic conditions were as follows: chromatographic column: C18, 250 mm long, 4.6 mm inner diameter, 5 μm particle size, or equivalent performance; column temperature: 25 °C; injection volume: 20 µL; the mobile phase: triethylamine phosphate solution + methanol = 95 + 5 mixed, filtered by 0.45 μm filter membrane, ultrasonic degassing; flow rate: 1 mL/min; the detection wavelength was 254 nm. The calculation method was as follows:

IMP (%) = (C × V)/(m × 10^6^)

C: The concentration of IMP (µg/mL) in the solution to be measured calculated by the standard curve.
V: The final volume of the sample (mL).
m: Weighed mass of the raw meat sample (g).

The standard curve range was 0.02–0.16 mg/mL (or 0–125 µg/mL), and the linear correlation coefficient R^2^ ≥ 0.999.

### 2.9. Determination of Amino Acids and Fatty Acids in Pork

The amino acids were determined using the automatic amino acid analyzer LA8080 (Hitachi, Ltd., Tokyo, Japan). The detection steps were performed as follows: An appropriate amount of evenly mixed sample was weighed, and 10–15 mL of 6 mol/L hydrochloric acid solution was added to the hydrolysis tube. The hydrolysis tube was placed in a refrigerant, frozen for 3–5 min, with nitrogen-filled protection, and the bottle cap was tightened. The hydrolysis tube was placed in an electrothermal blast incubator at 110 °C ± 1 °C for hydrolysis for 22 h and taken out and cooled to room temperature. The hydrolysis tube was opened, and the hydrolysate was filtered into a 50 mL volumetric flask. The hydrolysis tube was rinsed with a small amount of water for several times. The washing solution was transferred into the same 50 mL volumetric flask and finally diluted with water to the scale and shaken well. Then, 1.0 mL of the filtrate was pipetted into a 15 mL test tube, evaporated to dryness under reduced pressure at 40 °C, mixed with 1.0 mL of pH = 2.2 sodium citrate buffer solution, oscillated after mixing, filtered through a 0.22 μm filter membrane, and analyzed on the machine. The calculation method was as follows:

Amino Acid Content (g/kg) = (C × V)/m

C: The concentration of AA (g/mL) in the solution to be measured calculated by the standard curve.
V: The final volume of the sample (mL).
m: Weighed mass of the raw meat sample (kg).

The standard curve range was 0.1–50 μg/mL, and the linear correlation coefficient R^2^ ≥ 0.995.

The fatty acid contents were determined using the Agilent 7890A Gas Chromatograph (Agilent, Santa Clara, CA, USA), and the detection steps were performed as follows:

(1) Lipid extraction: An appropriate amount of sample was weighed into a glass centrifuge tube. A chloroform–methanol mixture (2:1, *v*/*v*) was added to fully immerse the sample. The mixture was sonicated or mechanically agitated (30 min at 25 °C) in a fume hood to enhance lipid dissolution. The supernatant was collected after centrifugation at 4000 rpm for 10 min. The extract was transferred to a nitrogen evaporator or rotary evaporator and concentrated to near-dryness under inert nitrogen gas to prevent fatty acid oxidation. The residue was reconstituted in 1 mL of n-hexane for subsequent derivatization.

(2) Saponification and methyl esterification: The lipid extract was supplemented with 2 mL of 2% (*w*/*v*) methanolic sodium hydroxide solution and heated at 85 °C in a thermostatic water bath for 30 min to hydrolyze triglycerides into free fatty acids. Following saponification, 3 mL of 14% boron trifluoride–methanol reagent was added to the mixture. The reaction was maintained at 85 °C under reflux conditions for 30 min to convert free fatty acids into fatty acid methyl esters (FAMEs). After cooling to ambient temperature (25 ± 2 °C), 1 mL of n-hexane was added for liquid–liquid extraction. The biphasic system was vortex-mixed for 2 min and allowed to stratify for 60 min.

(3) Extraction and purification: A 100 μL aliquot of the upper organic phase was diluted to 1 mL with HPLC-grade n-hexane and transferred to a 1.5 mL amber vial. The solution was filtered through a 0.45 μm nylon membrane using positive-pressure filtration to remove particulate contaminants. The filtrate was stored at 4 °C in light-protected amber vials and analyzed within 24 h to minimize degradation. Triplicate analyses were performed for each sample group.

(4) Instruments and methods: chromatographic column: CD-2560 (100 m × 0.25 mm × 0.20 μm); temperature program: 130 °C for 5 min, then increased to 240 °C at a rate of 4 °C/min for 30 min; injector temperature: 250 °C; carrier gas flow rate: 0.5 mL/min; split injection ratio: 10:1; detector: FID; detector temperature: 250 °C.

(5) Data calculation: The content of individual fatty acids in the sample was calculated using the following formula:

$$W=\frac{C\times V\times N}{m}\times k$$

W: Content of individual fatty acids in the sample, expressed in milligrams per kilogram (mg/kg).
C: Concentration of fatty acid methyl esters (FAMEs) in the test solution, in milligrams per liter (mg/L).
V: Final volume of the test solution, in milliliters (mL).
k: Conversion factor for transforming FAMEs to their corresponding free fatty acids (pre-determined from certified standards).
N: Dilution factor applied during sample preparation.
m: Weighed mass of the sample, in grams (g).

### 2.10. Determination of Volatile Compounds in Pork

After slaughter, 15 g of the longissimus thoracis muscle from the first and second lumbar vertebrae of the experimental pig were quickly stored in liquid nitrogen and transported by dry ice to Qingdao Kechuang Quality Testing Co., Ltd.(Qingdao, China) for analysis. The volatile compounds in fresh meat were separated and identified using headspace microextraction (SPME) pretreatment and gas chromatography–mass spectrometry (GC-MS), following the method provided by Wu et al. [19]. The detection steps were performed as follows: First, the frozen meat samples were thawed at room temperature. After homogenization, 5 g of meat samples was accurately weighed and placed in a 15 mL extraction bottle (not exceeding 1/4 of the extraction bottle), the cap was tightened, and then the bottle was left in the headspace at 85 °C for 40 min. An SPME needle was used (aging at 250 °C for 10 min before use, cooling to room temperature, and then sequentially washing with methanol, ethanol, ether, n-hexane, deionized water, and methanol) for extraction for 20 min. The fiber was manually desorbed in the GC injection port for 5 min at 240 °C in splitless mode.

Chromatographic separation was achieved using a TG-5MS capillary column (30 m × 0.25 mm × 0.25 μm) with helium carrier gas at a constant flow rate of 1 mL/min. The oven temperature program was initiated at 50 °C (2 min hold), ramped at 4 °C/min to 240 °C, and maintained for 5 min [20]. Mass spectrometric detection utilized electron ionization (70 eV) with ion source and transfer line temperatures set to 250 °C. Full-scan data acquisition spanned *m*/*z* 40–450 at 0.3 s intervals, with detector voltage maintained at 1400 V [21].

Quality assurance measures included daily calibration with perfluorotributylamine (PFTBA), triplicate solvent blanks analyzed every 10 samples, and verification of fiber performance via n-alkane retention time consistency (RSD < 5%). Compound identification combined AMDIS 2.72 deconvolution with NIST 14 library matching (similarity ≥ 80%), while quantification employed external ethyl ester calibration curves (0.1–100 ng/g).

### 2.11. Data Processing and Analysis

All experimental data were preprocessed using Microsoft Excel 2010 (Microsoft Corp., Redmond, WA, USA) prior to statistical analysis. Statistical significance was determined using SPSS Statistics 22.0 (IBM Corp., Armonk, NY, USA) through one-way analysis of variance (ANOVA) with post hoc Duncan’s multiple range test for intergroup comparisons. If *p* < 0.05, the difference was significant, and if 0.05 < *p* < 0.10, the difference was not significant, which was a trend. The results of the calculations are presented as mean and standard deviation.

## 3. Results

### 3.1. Analysis of Growth Performance of Bamei Pigs

As shown in Table 2, in terms of growth performance, there was no significant difference in FBW, ADFI, ADG, or FCR among the three groups, but the value of FBW and ADG in group II tended to increase (0.05 < *p* < 0.10), and the value of FCR in group II tended to decrease (*p* = 0.058).

### 3.2. Analysis of Slaughter Performance of Bamei Pigs

As shown in Table 3, the backfat thickness of the control group was significantly higher than that of the other two groups (*p* = 0.016). In terms of the pH of gastric contents after slaughter, compared with the control group, the pH of group II decreased by 43.04% (*p* = 0.045). There was no significant difference between other indicators.

### 3.3. Analysis of Longissimus Thoracis Muscle Meat Quality of Bamei Pigs

From Table 4, it can be seen that compared with the control group, the marble score of group I decreased by 13.61% (*p* < 0.05), while group II increased by 0.52% (*p* < 0.05). Low-protein diets had no significant effect on L*, a*, b*, or pH values between groups, but b_24h_* had a decreasing trend (*p* = 0.051).

### 3.4. Analysis of Longissimus Thoracis Muscle Nutrient Content of Bamei Pigs

From Table 5, it can be seen that compared with the control group, there was no significant difference in muscle nutrients composition between the groups.

### 3.5. Analysis of Longissimus Thoracis Muscle Amino Acids of Bamei Pigs

As shown in Table 6, in the control group, the Cystine content was significantly lower compared with group I and group II (*p* < 0.05). In group I, the content of Methionine, Serine, and Glycine were significantly higher compared with the control group and group II (*p* < 0.05), and there was no significant difference between other amino acids.

### 3.6. Analysis of Longissimus Thoracis Muscle Fatty Acids of Bamei Pigs

According to Table 7, a total of seven kinds of SFA, five kinds of MUFA, and seven kinds of PUFA were detected. Compared with the control group, the values of decanoic acid, tetradecenoic acid, and cis-11-eicosenoic acid in group II were significantly increased (*p* < 0.05), the values of erucic acid in groups I were significantly increased (*p* < 0.05), but the value of stearic acid was significantly decreased (*p* = 0.026). There was no difference in other indicators among the three groups.

### 3.7. Analysis of Volatile Compounds in Longissimus Thoracis Muscle of Bamei Pig

Based on the total ion flow chromatography of volatile compounds, the detection results of volatile compounds in the longissimus thoracis muscles of Bamei pigs are shown in Table 8. A total of 186, 200, and 194 volatile compounds were detected in the control group, group I, and group II. The majority of flavor compounds measured in this experiment were alkanes, followed by acids, aldehydes, and alcohols. As the basic dietary protein level of Bamei pigs decreased, the content of aldehydes and alkanes increased. From the results of this experiment, the effect of dietary protein level on volatile compounds in Bamei pig muscles was not significant, but it can increase the content and types of aldehydes and alkanes in volatile compounds in Bamei pig muscles.

### 3.8. Analysis of Relative Aontent of Main Volatile Compounds in Longissimus Thoracis Muscle of Bamei Pig

The relative content of the main volatile compounds in the longissimus thoracis muscles of Bamei pigs is shown in Table 9. The Hexanal, Heptaldehyde, Benzaldehide, E-2-Octenal, 2,3-Octanedione, and 2-Pentylfuran contents of group II were significantly higher than those in the control group and group I (*p* < 0.05), while the content of Phenylacetaldehyde in group I tended to be higher than that in the other two groups (*p* = 0.055). The 2-Heptanone and Limonene contents in the control group were significantly higher than those in the other two groups (*p* = 0.042).

## 4. Discussion

### 4.1. Effects of Dietary Protein Levels on Growth Performance of Bamei Pigs

According to the results of this experiment, it was found that the change in dietary protein level had no significant effect on the growth performance of Bamei pigs, but there was a tendency to increase FBW and ADG and decrease FCR, which was consistent with the results of Plandini et al. [22]. Studies have shown that low-protein amino-acid-balanced diets can improve the production performance of fattening pigs [23], but this result was affected by the type and content of the EAA added [24]. Studies have found that the diet of sows in the medium protein group can significantly improve the reproductive performance of pregnant sows and achieve the best economic benefits [25]. A study by Yin et al. [26] on low-protein diets balanced with four AAs (Lys, Met, Thr, and Trp) in piglets, growing pigs, and finishing pigs showed that compared with the high-protein group, the medium-protein group diet had no significant effect on the feed intake and body weight of piglets, growing pigs, and finishing pigs, while the low-protein group diet significantly inhibited the feed intake and body weight of piglets, growing pigs, and finishing pigs, indicating that the decrease in feed intake and body weight may be mainly related to limitations in protein and amino acid levels in the diet [27]. Previous studies have shown that tryptophan has a regulatory effect on pig feed intake, and leucine significantly reduces pig weight and feed intake [28,29]. AA supplementation can only replace the effect of protein on pigs in a small range. Qin et al. [30] showed that reducing the protein level in pig diets from 18% to 14%, while adding four limiting AAs, lysine, methionine, tryptophan, and threonine, did not affect pig growth performance. However, reducing the protein level in the diet to below 14% would hinder pig growth, even if the limiting amino acids were added; this may be because a decrease in dietary CP levels beyond a certain threshold means that adding only four EAAs is insufficient to meet the body’s demand for amino acids. The more dietary CP levels decrease, the more types of limiting AAs there are, leading to an imbalance of EAAs in low-protein diets. In this study, there was no significant difference in the effect of dietary protein levels on the production performance of Bamei pigs, but there was a trend towards promoting production performance.

### 4.2. Effect of Dietary Protein Levels on Slaughter Performance of Bamei Pigs

The influence of dietary CP levels on porcine slaughter performance predominantly manifests through alterations in lean meat percentage and backfat thickness [31,32]. Current scientific consensus indicates that when SID EAAs and energy requirements are adequately met, dietary protein content exerts minimal effects on carcass characteristics [22,33,34,35,36]. This principle was corroborated by multiple studies demonstrating that a strategic reduction in CP levels with concomitant EAA supplementation maintains slaughter performance parameters [37]. Notably, this study revealed that moderate CP levels significantly enhanced backfat thickness in Bamei pigs (*p* < 0.05), aligned with observations by Maeda et al. [38] and Chen [39]. This phenotypic response contrasted with reports of increased backfat deposition in conventional breeds under low-protein regimens, suggesting genetic variations in breed-specific responses to protein modulation [14,40,41,42]. The absence of significant effects on visceral organ weights in this study aligned with previous findings in commercial fattening pigs [43], while the observed growth performance improvements at 18.0% CP levels parallel findings in Rongchang pigs [44]. In summary, while dietary CP levels exerted varying effects on slaughter performance, the maintenance of balanced SID amino acids remains paramount. The observed differences across studies can largely be attributed to breed-specific nutritional requirements and metabolic efficiencies, emphasizing the need for tailored dietary strategies in swine production. These findings contribute to a deeper understanding of protein utilization in pigs and provide valuable insights for optimizing feed formulations to enhance production efficiency and meat quality.

### 4.3. Effects of Dietary Protein Levels on the Quality and Muscle Nutrient Content of Bamei Pork

The effects of dietary protein levels on meat quality of livestock and poultry have different results. In this study, there was no significant difference in muscle nutrients among the treatment groups, which was consistent with the results of Jiang et al. [45] and Alfaia et al. [46]. As an important indicator of meat freshness, IMP has the characteristics of determining muscle umami [47]. In this study, there was no significant difference in the effect of dietary protein levels on the IMP of Bamei pigs. Meat color is a key visual indicator of meat quality, and higher a* values are associated with better meat quality because they reflect the concentration of myoglobin in muscle tissue [48,49,50]. Studies have demonstrated that AA-balanced low-protein diets can significantly reduce the shear force of the longissimus thoracis muscle in fattening pigs, thereby improving tenderness [51,52]. Additionally, low-protein diets have been shown to enhance muscle marbling scores and tenderness without significantly impacting meat color or pH [53,54,55]. These findings suggest that protein levels and AA balance are critical factors influencing the texture and sensory attributes of meat. In this study, a reduction in dietary protein levels in Bamei pigs led to increased muscle shear strength, reduced cooking loss, and improved marbling. Furthermore, the post-slaughter decline in meat pH and the increase in muscle water content were attenuated. The observed improvements in meat quality can be attributed to the enhanced crude ash and inosinic acid content, which positively influence crude protein and fat levels. These results highlight the potential of low-protein diets to enhance the sensory and physicochemical properties of Bamei pork. In summary, while the effects of dietary protein levels on meat quality vary across species and experimental conditions, in this study, there was no significant difference in the effect of dietary protein levels on the meat quality parameters of Bamei pigs. These insights contribute to a deeper understanding of the relationship between dietary protein and meat quality, providing valuable implications for optimizing feed formulations to enhance the palatability and marketability of pork products.

### 4.4. Effect of Dietary Protein Levels on Muscle Amino Acids in Bamei Pigs

Aas are fundamental building blocks of proteins, which play a pivotal role in determining the nutritional value and quality of meat products. Their content and composition are critical indicators of meat quality, influencing flavor, texture, and nutritional attributes. AAs can be categorized into four groups: FAAs, SAAs, AAAs, and BAAs [56]. Previous studies have demonstrated that reducing dietary CP levels can significantly decrease the concentration of AAs in pig muscles [57,58,59]. In this study, no significant differences were observed in the SAA, FAA, and AAA contents across the experimental groups. This suggests that reducing CP levels within the tested range did not compromise these specific AA categories. Notably, the flavor of pork is largely derived from ribose and cystine [60]. In this study, the cystine content in the muscle of the experimental group was significantly higher than that of the control group (*p* < 0.001), indicating that the low-protein dietary group exhibited enhanced meat flavor. Additionally, group I demonstrated higher levels of TAAs, EAAs, and SAAs compared to the other groups. In summary, this study highlighted the potential of low-protein diets in improving meat quality in Bamei pigs by enhancing AA content, particularly cystine, which plays a critical role in flavor development. However, the precise relationship between dietary protein levels, AA supplementation, and meat quality remains an area for further exploration. These findings contribute to a deeper understanding of dietary strategies aimed at optimizing pork quality and nutritional value.

### 4.5. Effect of Dietary Protein Levels on Muscle Fatty Acids in Bamei Pigs

Fatty acids, which are classified into UFA and SFA, play a critical role in determining meat quality and flavor. UFA is further categorized into MUFA and PUFA [61]. SFA includes octanoic acid, decanoic acid, lauric acid, myristic acid, palmitic acid, stearic acid, arachidic acid, etc., while excessive SFA consumption is associated with health risks such as arteriosclerosis [62]. UFA is beneficial for human health, exhibiting anticancer, lipid-lowering, and cardiovascular disease prevention properties [63,64]. The composition of fatty acids is a key determinant of meat quality, as during heating, fat undergoes the Maillard reaction, generating desirable aromas and enhancing flavor [65]. Notably, the content of UFAs directly influenced meat flavor, with PUFAs being the primary contributors to pork flavor [66]. Previous studies have demonstrated that reducing dietary protein levels by 2.5% under conditions of essential amino acid balance significantly impacts the meat quality of fattened pigs [67,68]. Specifically, low-protein diets have been shown to reduce linolenic acid levels while increasing arachidonic acid levels in muscle [69]. Additionally, the content of palmitic acid, palmitoleic acid, and oleic acid in muscle has been identified as an important indicator of meat quality improvement [70]. In this study, compared to the control group, group II exhibited significantly higher levels of decanoic acid, myristic acid, and cis-11-eicosenoic acid (*p* < 0.05). Similarly, erucic acid and the PUFA/TFA ratio were significantly increased in groups I and II (*p* < 0.05), while the stearic acid content was significantly reduced (*p* = 0.026). These results indicate that as dietary protein levels decreased, the UFA/TFA, MUFA/TFA, and PUFA/TFA ratios in Bamei pig muscles increased, leading to an improved fatty acid composition and, consequently, enhanced meat quality.

### 4.6. Effects of Dietary Protein Levels on the Types and Content of Volatile Compounds in the Muscles of Bamei Pigs

The flavor of pork is largely influenced by volatile compounds, particularly aldehydes, alcohols, and ketones, which undergo significant changes during processing and storage, affecting the sensory attributes of meat [71,72]. In this study, the experimental groups exhibited a higher diversity and relative content of aldehyde compounds in muscle compared to the control group, suggesting a potential enhancement in meat flavor. Alcohol compounds also play a critical role in meat flavor [73]; however, ester compounds were found to have a minimal impact on pork flavor [74]. The volatile compounds measured in this study were predominantly alkanes, followed by acids, aldehydes, and alcohols. Although alkanes, including hydrocarbons such as alkanes and olefins, contribute little directly to meat flavor, they serve as precursors that can be oxidized and decomposed into flavor-active compounds such as aldehydes, ketones, and small amounts of esters, thereby enhancing the overall flavor profile of meat products [75]. In this study, groups I and II exhibited a higher number of alkanes compared to the control group, indicating a potential for greater flavor development through oxidation processes. Among the flavor-active compounds, 2,3-Octanedione, a ketone, was significantly more abundant in group II than in the other groups (*p* < 0.01), highlighting its potential role in flavor enhancement. Aldehydes are critical contributors to meat flavor [75,76]. Specifically, Hexanal and E-2-Octenal were significantly higher in group II than in the other groups (*p* < 0.05), while Heptaldehyde was significantly elevated in group II compared to the control group (*p* = 0.025). This study shows that aldehydes are key contributors to the improved flavor profile observed in the low-protein diet groups. Alcohols, another important class of flavor compounds, also exhibited notable changes [77]. The 14.0% CP group demonstrated an increased number of volatile compounds and higher concentrations of major volatile compounds compared to the control group. This indicates that the low-protein diet not only enhanced the diversity of flavor-active compounds but also positively influenced the overall flavor of the meat. In conclusion, this study demonstrates that reducing dietary protein levels can significantly alter the volatile compound profile of pork, leading to improved flavor attributes. The increased abundance of aldehydes, ketones, and alcohols in the low-protein diet groups highlights the potential of such dietary strategies to enhance meat flavor. These findings provide valuable insights into the mechanisms underlying flavor development in pork and underscore the importance of optimizing dietary protein levels to achieve superior meat quality. Further research is warranted to explore the specific pathways through which low-protein diets modulate volatile compound formation and flavor characteristics.

## 5. Conclusions

In this study, reducing the dietary protein level of Bamei pigs and supplementing four amino acids had no significant effect on production performance and meat quality, but could significantly reduce backfat thickness. Furthermore, the low-protein level group had better muscle AA composition and more types and contents of muscle flavor compounds. Based on the results of this study, the most suitable crude protein level for the fattening period of Bamei pigs was 14.0%. At the same time, exploring the effect of low-protein diet on the production performance and meat quality of pigs can better improve breeding efficiency and promote the green development of pig breeding.

## Figures and Tables

**Table 1:** Composition and nutrient content of experimental diets (% of DM basis).

Raw Material	Control Group	Group I	Group II
Corn	63.50	68.00	72.00
Soybean meal	18.30	12.90	7.20
Wheat bran	5.00	5.00	5.00
Alfalfa meal	5.00	6.50	9.00
Bentonite	4.00	2.90	1.60
Soybean oil	1.50	1.70	2.00
Allzyme ^①^	0.10	0.10	0.10
Lysine	0.09	0.23	0.37
Methionine	-	0.03	0.05
Threonine	-	0.08	0.15
Tryptophan	-	0.03	0.05
CaCO_3_	0.51	0.46	0.30
CaHPO_4_	1.15	1.22	1.33
vit. and mineral mix ^②^	0.50	0.50	0.50
NaCl	0.35	0.35	0.35
Total	100.00	100.00	100.00
Nutrient content ^③^			
DE/(MJ/kg)	13.14	13.13	13.11
CP	16.00%	14.00%	12.00%
CF	3.22	3.50	4.02
Ca	0.60	0.61	0.60
TP	0.55	0.55	0.55
Na	0.16	0.16	0.17
Cl	0.27	0.27	0.28
SID Lys	0.86	0.86	0.86
SID Met	0.26	0.26	0.26
SID Thr	0.59	0.59	0.59
SID Trp	0.19	0.19	0.19

^①^ Allzyme is a multi-enzyme complex produced by Aspergillus niger through improved solid-state fermentation technology, which mainly contains phytase, glucanase, cellulase, pectinase, amylase, protease, and xylanase. ^②^ The premix provided the following per kg of diets: Fe 64.00 mg, Zn 71.00 mg, Mn 35.00 mg, Cu 17.00 mg, Se 0.36 mg, I 0.64 mg, Vitamin A 790 IU, Vitamin D3 135 IU, Vitamin E 55.00 mg, thiamine (Vitamin B1) 2.20 mg, riboflavin (Vitamin B2) 2.50 mg, biotin 0.05 mg, folic acid 0.35 mg, nicotinic acid 29.00 mg, calcium pantothenate 27.00 mg, Vitamin B6 0.09 mg, Vitamin B12 1.00 mg, choline chloride 5000.00 mg, flavoring agent 3000.00 mg, sweetening agent 3000.00 mg, phytase 4000.00 mg, Lys 30,000.00 mg, and Try 2000.00 mg. ^③^ Nutrient levels were calculated on the basis of DM. Lys: Lysine, Met: Methionine, Thr: Threonine, Trp: Tryptophan, CF: Crude Fiber, TP: Total Phosphorus.

**Table 2:** Effects of dietary protein levels on growth performance of Bamei pigs.

Items	Control Group	Group I	Group II	*p*-Value
IBW/kg	50.85 ± 2.12	51.05 ± 2.01	51.20 ± 1.58	0.125
FBW/kg	104.45 ± 3.60	102.95 ± 3.08	108.67 ± 2.29	0.085
ADFI/kg·day^−1^	2.30 ± 0.26	2.32 ± 0.14	2.37 ± 0.16	0.110
ADG/kg·day^−1^	0.77 ± 0.05	0.74 ± 0.01	0.82 ± 0.02	0.062
FCR/kg feed·kg gain^−1^	2.99 ± 0.61	3.14 ± 0.56	2.89 ± 0.46	0.058

Note: Values are expressed as the mean and standard error of the mean (SEM). *n* = 12.

**Table 3:** Effects of dietary protein levels on slaughter performance of Bamei pigs.

Item	Control Group	Group I	Group II	*p*-Value
Carcass weight/kg	71.84 ± 1.46	71.56 ± 0.90	76.31 ± 1.46	0.105
Dressing percentage/%	68.78 ± 2.30	69.51 ± 1.81	70.22 ± 1.17	0.456
Backfat thickness/cm	5.54 ± 0.68 ^a^	3.38 ± 1.01 ^b^	3.34 ± 0.88 ^b^	0.016
Skin thickness/mm	2.88 ± 0.13	3.30 ± 2.57	3.25 ± 1.14	0.088
Carcass length/cm	99.20 ± 4.30	102.19 ± 9.00	98.11 ± 10.92	0.232
Loin eye area/cm^2^	36.08 ± 7.15	19.60 ± 10.27	34.99 ± 8.62	0.844
Hind leg weight/kg	8.71 ± 2.58	9.93 ± 1.18	8.75 ± 2.59	0.112
Heart weight/kg	0.30 ± 0.00	0.28 ± 0.06	0.33 ± 0.10	0.762
Liver weight/kg	1.38 ± 0.03	1.08 ± 0.10	1.08 ± 0.70	0.684
Spleen weight/kg	0.18 ± 0.03	0.20 ± 0.05	0.14 ± 0.06	0.621
Lung weight/kg	0.75 ± 0.01	0.67 ± 0.03	0.77 ± 0.10	0.301
Kidney weight/kg	0.33 ± 0.03	0.26 ± 0.06	0.35 ± 0.09	0.193
Stomach weight/kg	0.93 ± 0.08	0.85 ± 0.01	0.92 ± 0.13	0.274
Gastric contents pH	6.18 ± 0.57 ^a^	5.24 ± 0.89 ^ab^	3.52 ± 1.28 ^b^	0.045
Cecal contents pH	6.39 ± 0.43	6.43 ± 0.04	5.90 ± 0.39	0.324
Jejunal contents pH	6.84 ± 0.20	6.79 ± 0.21	6.67 ± 0.45	0.186

Note: Values are expressed as the mean and standard error of the mean (SEM). *n* = 6. a,b: means with different lowercase letters differ significantly among the four treatments (*p* < 0.05); ab: means in a row sharing the same superscript do not differ (*p* > 0.05).

**Table 4:** Effect of dietary protein levels on longissimus thoracis muscle meat quality in Bamei pigs.

Item	Control Group	Group I	Group II	*p*-Value
Shear force/N	46.18 ± 0.03	48.35 ± 1.52	47.71 ± 2.52	0.294
Filtration rate/%	4.89 ± 0.31	4.17 ± 0.81	6.07 ± 1.44	0.062
Cooking loss/%	33.94 ± 1.29	34.89 ± 3.07	34.16 ± 3.70	0.058
Marbling score	3.82 ± 0.14 ^a^	3.30 ± 0.30 ^b^	3.84 ± 0.14 ^a^	0.024
L_45 min_	42.05 ± 0.45	39.14 ± 3.41	41.85 ± 2.23	0.900
L_24 h_	48.59 ± 2.89	44.98 ± 4.00	49.38 ± 2.23	0.141
a_45 min_	4.02 ± 0.32	5.74 ± 1.65	4.08 ± 1.39	0.147
a_24 h_	9.54 ± 0.24	8.03 ± 2.13	8.41 ± 1.38	0.156
b_45 min_	11.88 ± 0.85	10.50 ± 1.29	12.42 ± 0.46	0.273
b_24 h_	16.34 ± 0.84	14.82 ± 1.49	16.18 ± 0.05	0.051
pH_45 min_	6.27 ± 0.03	6.22 ± 0.22	6.15 ± 0.42	0.119
pH_24 h_	5.53 ± 0.15	5.49 ± 0.17	5.56 ± 0.11	0.620
pH_72 h_	5.52 ± 0.05	5.41 ± 0.20	5.50 ± 0.09	0.367

Note: Values are expressed as the mean and standard error of the mean (SEM). *n* = 6. a,b: means with different lowercase letters differ significantly among the four treatments (*p* < 0.05); ab: means in a row sharing the same superscript do not differ (*p* > 0.05). The marble score was divided into 10 grades, from 1.0 to 10.0, of which 10.0 was the clearest and richest.

**Table 5:** Effect of dietary protein levels on nutrient content in longissimus thoracis muscle of Bamei pigs (Fresh meat).

Compound (%)	Control Group	Group I	Group II	*p*-Value
Moisture	66.25 ± 1.55	68.27 ± 2.96	68.47 ± 1.60	0.146
Crude protein (CP)	22.65 ± 0.35	23.30 ± 1.18	22.17 ± 2.27	0.402
Crude fat (EE)	6.80 ± 0.50	6.27 ± 1.51	6.03 ± 1.11	0.447
Ash	1.10 ± 0.03	1.11 ± 0.06	1.15 ± 0.05	0.253
Inosinc acid (IMP)	0.30 ± 0.03	0.28 ± 0.02	0.27 ± 0.01	0.129

Note: Values are expressed as the mean and standard error of the mean (SEM). *n* = 6.

**Table 6:** Effects of dietary protein levels on amino acids in longissimus thoracis muscle of Bamei pigs.

Amino Acid (g/kg)	Control Group	Group I	Group II	*p*-Value
Threonine	7.88 ± 0.07	8.98 ± 0.87	7.59 ± 0.70	0.085
Valine	9.56 ± 0.22	13.28 ± 5.01	9.01 ± 0.77	0.233
Methionine	4.71 ± 0.03 ^b^	11.33 ± 1.93 ^a^	3.89 ± 1.53 ^b^	0.016
Isoleucine	10.30 ± 0.17	10.06 ± 1.81	9.66 ± 0.91	0.789
Leucine	17.38 ± 0.28	17.33 ± 2.92	16.77 ± 1.38	0.899
Phenylalanine	8.69 ± 0.12	11.48 ± 3.52	8.38 ± 0.64	0.210
Lysine	20.17 ± 0.04	17.43 ± 8.45	19.78 ± 1.80	0.776
Cystine	7.41 ± 0.01 ^b^	8.93 ± 0.37 ^a^	9.27 ± 0.14 ^a^	<0.001
Tyrosine	9.71 ± 0.15	13.76 ± 5.71	9.41 ± 0.99	0.282
Histidine	14.39 ± 0.29	12.04 ± 7.72	13.05 ± 2.33	0.830
Serine	8.48 ± 0.17 ^b^	9.38 ± 0.11 ^a^	8.36 ± 0.66 ^b^	0.039
Arginine	14.60 ± 0.14	13.52 ± 5.31	14.99 ± 0.86	0.841
Aspartic acid	19.28 ± 0.36	20.70 ± 0.57	18.69 ± 1.62	0.118
Glycine	8.46 ± 0.06 ^b^	9.06 ± 0.40 ^a^	8.18 ± 0.18 ^b^	0.015
Glutamic acid	26.83 ± 0.08	22.00 ± 11.63	26.07 ± 2.15	0.666
Alanine	20.21 ± 0.26	20.32 ± 1.51	19.46 ± 1.42	0.811
Proline	8.74 ± 0.05	9.08 ± 0.44	8.55 ± 0.32	0.189
TAA	216.80 ± 3.02	228.68 ± 13.34	211.12 ± 17.42	0.160
EAA	104.79 ± 1.86	123.21 ± 16.04	102.80 ± 9.38	0.055
FAA	69.17 ± 0.58	65.28 ± 17.16	67.93 ± 9.00	0.895
SAA	73.94 ± 1.25	74.25 ± 10.22	71.93 ± 5.04	0.899
AAA	60.50 ± 0.57	57.74 ± 19.84	57.81 ± 6.07	0.845
BAA	89.34 ± 1.50	102.81 ± 9.87	85.16 ± 8.50	0.065
Glutamate/TAA (%)	12.38 ± 1.12	9.44 ± 2.58	12.35 ± 0.86	0.071
EAA/TAA (%)	48.33 ± 5.84	54.27 ± 3.25	48.67 ± 6.25	0.088
FAA/TAA (%)	31.70 ± 3.68	28.32 ± 2.56	32.21 ± 2.77	0.074

Note: Values are expressed as the mean and standard error of the mean (SEM). *n* = 6. a,b: means with different lowercase letters differ significantly among the four treatments (*p* < 0.05); ab: means in a row sharing the same superscript do not differ (*p* > 0.05). Essential amino acid (EAA) = sum of valine, isoleucine, leucine, phenylalanine, methionine, tryptophan, threonine, and lysine; flavor amino acid (FAA) = sum of aspartate, glycine, alanine, phenylalanine, and glutamic acid; sweet amino acids (SAAs) = sum of threonine, lysine, glycine, serine, alanine, and proline; acid amino acids (AAAs) = sum of glutamate, aspartic acid, and histidine; bitter amino acid (BAA) = sum of histidine, valine, methionine, isoleucine, leucine, phenylalanine, tyrosine, tryptophan, and arginine; total amino acid (TAA) = total amino acids.

**Table 7:** Effects of dietary protein levels on fatty acids in longissimus thoracis muscle of Bamei pigs.

Group	Fatty Acid (mg/kg)	Control Group	Group I	Group II	*p*-Value
Saturated Fatty Acid (SFA)	C10:0 Decanoic Acid	46.00 ± 2.01 ^c^	78.67 ± 28.11 ^b^	83.3 ± 19.14 ^a^	0.012
	C12:0 Lauric acid	59.50 ± 0.50	66.67 ± 21.94	65.33 ± 21.59	0.125
	C14:0 Myristic acid	1084.00 ± 2.00	944.67 ± 357.03	1009.22 ± 264.59	0.158
	C16:0 Palmitic acid	19,462.00 ± 814.00	15,961.67 ± 3968.80	15,925.33 ± 3934.64	0.213
	C17:0 Heptadecanoic acid	56.50 ± 13.50	84.67 ± 30.02	77.33 ± 13.05	0.241
	C18:0 Stearic acid	8745.50 ± 338.50 ^a^	6408.33 ± 1134.80 ^b^	6631.00 ± 1035.83 ^b^	0.026
	C20:0 Arachidic acid	158.50 ± 2.50	122.00 ± 43.59	113.33 ± 37.00	0.153
Monounsaturated Fatty Acid (MUFA)	C14:1 Tetradecenoic acid	22.50 ± 2.50 ^b^	22.00 ± 10.00 ^b^	40.00 ± 10.00 ^a^	<0.001
	C16:1 Palmitoleic acid	2840.50 ± 249.50	2591.33 ± 644.80	2878.67 ± 1131.18	0.136
	C18:1n9c Oleic acid	29,356.50 ± 2690.50	28,796.00 ± 8380.40	26,911.67 ± 3840.88	0.154
	C20:1 cis-11-eicosenoic acid	83.00 ± 19.00 ^c^	126.00 ± 67.54 ^b^	302.00 ± 347.62 ^a^	<0.001
	C22:1n9 Erucic acid	18.00 ± 0.01 ^b^	37.33 ± 15.01 ^a^	28.00 ± 4.00 ^ab^	0.021
Polyunsaturated Fatty Acid (PUFA)	C18:2n6c Linoleic acid	2083.00 ± 425.00	3345.00 ± 1624.84	2680.00 ± 411.14	0.223
	C18:3n3 α-linolenic acid	472.00 ± 7.00	571.33 ± 254.34	319.00 ± 176.37	0.210
	C18:3n6 γ-linolenic acid	18.00 ± 8.00	26.67 ± 10.07	18.00 ± 0.01	0.123
	C20:2 cis-11,14-eicosadienoic acid	104.00 ± 22.00	171.67 ± 81.16	127.00 ± 26.00	0.214
	C20:3n3 cis-11,14,17-eicosatrienoic acid	30.00 ± 11.00	55.33 ± 24.58	65.67 ± 63.51	0.244
	C20:3n6 cis-8,11,14-eicosatrienoic acid	47.50 ± 21.50	69.67 ± 22.03	65.00 ± 16.46	0.112
	C20:4n6 Arachidonic acid	315.50 ± 190.50	375.00 ± 178.60	316.33 ± 139.59	0.212
Total Fatty Acid (TFA)		65,022.50 ± 4813.62	59,904.33 ± 14,533.43	57,711.33 ± 10,378.71	0.118
SFA/TFA (%)		45.54 ± 5.78	39.51 ± 10.41	41.42 ± 8.95	0.211
UFA/TFA (%)		54.43 ± 12.21	60.41 ± 14.21	58.48 ± 16.01	0.115
MUFA/TFA (%)		49.71 ± 8.66	52.71 ± 9.45	52.26 ± 8.78	0.089
PUFA/TFA (%)		4.72 ± 2.41 ^b^	7.70 ± 1.89 ^a^	6.22 ± 1.11 ^a^	0.002

Note: Values are expressed as the mean and standard error of the mean (SEM). *n* = 6. a,b,c: means with different lowercase letters differ significantly among the four treatments (*p* < 0.05), ab: means in a row sharing the same superscript do not differ (*p* > 0.05).

**Table 8:** Effects of dietary protein levels on volatile compounds in longissimus thoracis muscle of Bamei pigs.

Compound	Control Group	Group I	Group II
Phenolics	12	11	11
Aldehydes	28	32	29
Alcohols	24	32	26
Acids	16	16	17
Ketones	12	11	19
Sulfides	5	7	7
Alkanes	28	31	33
Benzenes	5	3	7
Furans	2	4	3
Thiazoles	4	5	1
Esters	45	44	33
Others	5	4	8
Total	186	200	194

**Table 9:** Effects of dietary protein level on the relative amount of main volatile compounds in longissimus thoracis muscle of Bamei pigs (%).

Compound Name (%)	Control Group	Group I	Group II	*p*-Value
Hexanal	4.43 ± 3.37 ^c^	7.56 ± 1.68 ^b^	11.33 ± 7.82 ^a^	<0.001
Heptaldehyde	0.45 ± 0.33 ^b^	0.84 ± 0.16 ^ab^	1.88 ± 1.00 ^a^	0.025
Phenylacetaldehyde	0.26 ± 0.21	0.95 ± 0.48	0.66 ± 0.40	0.055
Benzaldehyde	0.25 ± 0.43 ^b^	3.49 ± 1.59 ^a^	4.53 ± 0.46 ^a^	0.046
E-2-Octenal	0.27 ± 0.06 ^b^	0.59 ± 0.08 ^b^	1.39 ± 0.30 ^a^	0.039
Nonanal	3.40 ± 2.54	4.31 ± 0.65	6.68 ± 1.83	0.066
Amyl alcohol	0.24 ± 0.42 ^b^	0.33 ± 0.27 ^b^	0.76 ± 0.21 ^a^	0.029
Hexyl alcohol	0.33 ± 0.58	0.17 ± 0.02	0.27 ± 0.02	0.051
2-Heptanone	1.91 ± 0.04 ^a^	0.51 ± 0.02 ^b^	0.55 ± 0.03 ^b^	0.042
2,3-Octanedione	1.27 ± 0.04 ^c^	2.21 ± 0.71 ^b^	3.27 ± 0.01 ^a^	<0.001
Limonene	9.07 ± 0.06 ^a^	0.32 ± 0.15 ^b^	0.29 ± 0.14 ^b^	<0.001
2-Pentylfuran	0.22 ± 0.01 ^b^	0.55 ± 0.12 ^b^	1.44 ± 0.43 ^a^	0.036

Note: Values are expressed as the mean and standard error of the mean (SEM). *n* = 6. a,b,c: means with different lowercase letters differ significantly among the four treatments (*p* < 0.05); ab: means in a row sharing the same superscript do not differ (*p* > 0.05).

## Data Availability

The original contributions presented in the study are included in the article, further inquiries can be directed to the corresponding author.
